# Papoid in Gastro-Intestinal Disorders*Extract from article in *New York Medical Journal*, of July 30th.

**Published:** 1892-09

**Authors:** Frank Woodbury


### Selections and Abstracts.

### PAPOID IN GASTRO-INTESTINAL DISORDERS.*

BY DR. FRANK WOODBURY.

During the past year, having devoted considerable attention to the clinical application of papoid, especially in digestive disorders, I have had the satisfaction of witnessing a number of very interesting results, to which I wish briefly to direct attention. The successful application of physiological data must be my excuse for again directing attention to a remedy which has been studied by such eminent investigators as Wurtz, Bouchut, Finckler, Rossbach, Roy and Wittmach, and one furthermore, the physiological and therapeutical actions of which may be regarded as pretty fully established. I point out very briefly some of the clinical uses and the conditions of its successful employment.

The physiological actions of papoid as a digestive agent have been thoroughly established. It acts upon albuminoids, hydrating and converting them into peptones, as fully demonstrated by Herschell. It converts starch with great promptness, the ultimate product being maltose. It emulsifies fats. Herschell declares that it has a direct tonic action on the stomach, stimulates the secretion of gastric juice or pepsinogen. It acts at all temperatures, but attains its maxim activity at about 130 deg. F. In several important points it differs from pepsin. Papoid acts best in an alkaline solution, but also can act in fluids with an acid or neutral reaction. Pepsin requires an acid solution. Papoid is freely soluble and is most active when in concentrated form. Pepsin requires free dilution. Herschell also points out the greater digestive power possessed by papoid than either pepsin or pancreatine, and states "that it can be used when pepsin is contra-indicated or powerless." Papoid has no action upon living tissues, and is positively innocuous when swallowed in any

*Extract from article in *New York Medical Journal*, of July 30th.

quantity that is likely to be administered. Papoid is useful when digestion has been overtaxed, or when the secretion of gastric juice is absent or deficient. Experiments of my own and others have satisfied my mind of the remarkable digestive activity of papoid. In one of the experiments referred to, the constituents of a hearty dinner of bread, meat, potatoes, peas, mince-pie, and other substantials were placed in a test tube and treated with papoid and bicarbonate of sodium and a small amount of water. The result was very satisfactory indeed; the meat rapidly softened, and the other ingredients gradually disintegrated, forming a pultaceous mass, which finally separated into a grumous sediment and an overlying, albuminous, dark colored liquid. Papoid acts in an alkaline solution even better than in acid media. It is evident that it is specially useful where there is indigestion due to deficient secretion of gastric juice. In such cases, the administration of an alkaline solution of papoid favors gastric digestion, both directly and indirectly: First by digesting albuminates and softening masses of food. Secondly, by the action of the papoid in stimulating the secretion of the pepsin gland. It retards the fermentation of the undigested masses of food in the stomach, and prepares them for intestinal digestion. In the contrary case where the stomach contents poured into the duodenum are so acid that they prevent the action of the trypsin. Papoid prevents duodenal indigestion by taking the place of the pancreatic ferment. As Herschell says, it is obviously of no use to give pancreatine by the mouth, as it is at once destroyed by the acid of the stomach, and it is practically impossible to administer sufficient alkali to neutralize the excess of acid, and it would moreover be unwise, because it would stimulate still further the secretion of the acid. Papoid is of the greatest use here. In gastralgia, which often accompanies the condition just named, papoid with bi-carbonate of sodium gives immediate relief. It is also useful in irritable stomach, nausea and vomiting. In gastric catarrh, and the catarrhal conditions of the intestinal tract, popularly known as biliousness, papoid administered in hot water fifteen minutes before meals, cleanses off the mucus and places the mucous coat of the digestive organs in a good condition for secretion. It is useful in the treatment of irritative diarrhœa in young children, to whom it may be given in combination with salol or salicylate of bismuth. The use of papoid in treating disorders of the digestive organs may be summarized somewhat as follows:

1. In actual or relative deficiency of the gastric juice, or its constituents. (*a*) Diminished secretion of gastric juice as a whole. Apepsia; anæmia and deficient blood supply, wasting diseases. (*b*) Diminished proportion of pepsin; atonic dyspepsia, atrophy of the gastric tubules. (*c*) Dimunition of hydrochloric acid; achlorhydria, carcinoma. (*d*) Relative deficiency of gastric juice; overfeeding.

2. In gastric catarrh. (*a*) Where there is a tenacious mucus to be removed, thus enabling the food to come in contact with the mucous membrane. (*b*) Where there is impaired digestion.

3. In excessive secretion of acid. To prevent duodenal dyspepsia.

4. In gastralgia, irritable stomach, nausea or vomiting.

5. In intestinal disorders. (*a*) In constipation due to indigestion. (*b*) In diarrhœa, as a sedative. (*c*) In intestinal worms. (This claim the writer has not personally verified, but as the intestinal mucus, which shields the worms is removed by papoid, it is easily understood that their removal would naturally result after its administration.)

6. In infectious disorders of the intestinal tract. (*a*) Where there is abnormal fermentation by its antiseptic action which may be heightened by combination. (*b*) Where there are foreign substances present, its detergent effect may be utilized by cleaning out the debris from the intestinal contents by digestion.

7. In infantile indigestion; here papoid not only readily peptonizes cow's milk, but the resulting curds are also soft and flocculent, resembling those of breast milk.

The dose of papoid ordinarily, is one or two grains, but five grains or more may be used, the only objection being that of useless expense and waste, except where very prompt effects are desired.

